# Decreased Premotor Cortex Volume in Victims of Urban Violence with Posttraumatic Stress Disorder

**DOI:** 10.1371/journal.pone.0042560

**Published:** 2012-08-31

**Authors:** Vanessa Rocha-Rego, Mirtes G. Pereira, Leticia Oliveira, Mauro V. Mendlowicz, Adriana Fiszman, Carla Marques-Portella, William Berger, Carlton Chu, Mateus Joffily, Jorge Moll, Jair J. Mari, Ivan Figueira, Eliane Volchan

**Affiliations:** 1 Instituto de Biofísica Carlos Chagas Filho, Universidade Federal do Rio de Janeiro, Rio de Janeiro, Brazil; 2 Universidade Federal Fluminense, Niteroi, Brazil; 3 Instituto de Psiquiatria, Universidade Federal do Rio de Janeiro, Rio de Janeiro, Brazil; 4 University College London, London, United Kingdom; 5 D'Or Institute for Research and Education, Rio de Janeiro, Brazil; 6 Universidade Federal de São Paulo, São Paulo, Brazil; Royal Holloway, University of London, United Kingdom

## Abstract

**Background:**

Studies addressing posttraumatic stress disorder (PTSD) have demonstrated that PTSD patients exhibit structural abnormalities in brain regions that relate to stress regulation and fear responses, such as the hippocampus, amygdala, anterior cingulate cortex, and ventromedial prefrontal cortex. Premotor cortical areas are involved in preparing to respond to a threatening situation and in representing the peripersonal space. Urban violence is an important and pervasive cause of human suffering, especially in large urban centers in the developing world. Violent events, such as armed robbery, are very frequent in certain cities, and these episodes increase the risk of PTSD. Assaultive trauma is characterized by forceful invasion of the peripersonal space; therefore, could this traumatic event be associated with structural alteration of premotor areas in PTSD?

**Methodology/Principal Findings:**

Structural magnetic resonance imaging scans were acquired from a sample of individuals that had been exposed to urban violence. This sample consisted of 16 PTSD patients and 16 age- and gender-matched controls. Psychometric questionnaires differentiated PTSD patients from trauma-exposed controls with regard to PTSD symptoms, affective, and resilience predispositions. Voxel-based morphometric analysis revealed that, compared with controls, the PTSD patients presented significant reductions in gray matter volume in the ventral premotor cortex and in the pregenual anterior cingulate cortex.

**Conclusions:**

Volume reduction in the premotor cortex that is observed in victims of urban violence with PTSD may be associated with a disruption in the dynamical modulation of the safe space around the body. The finding that PTSD patients presented a smaller volume of pregenual anterior cingulate cortex is consistent with the results of other PTSD neuroimaging studies that investigated different types of traumatic events.

## Introduction

Post-Traumatic Stress Disorder (PTSD) is an anxiety disorder following an exposure to a traumatic event. The diagnostic criteria for PTSD require experiencing, witnessing, or being confronted with an event or events that involve actual or threatened death or serious injury, or a threat to the physical integrity of self or others (criterion A1). It also requires that the person experience intense fear, helplessness, or horror during the traumatic event (criterion A2). PTSD is characterized by a series of symptoms, including intrusions (e.g., nightmares or flashbacks), hyperarousal (e.g., insomnia or an exaggerated startle response), numbing (e.g., restricted affect or anhedonia), and avoidance of trauma-related stimuli. A diagnosis of PTSD requires these symptoms to last for a minimum of one month and disrupt the normal functioning of the patient [1]. Lifetime prevalence of PTSD in the general population of the United States was estimated at 7.8% [2]. PTSD follows a chronic course that causes patients to experience significant functional impairment and increase their usage of healthcare resources, resulting in substantial personal and societal costs [3], [4].

Over the past several years, neuroimaging studies of PTSD subjects have focused on elucidating the brain circuits that mediate this disorder [5]–[8]. Several PTSD studies have reported structural abnormalities in brain regions related to stress regulation and fear circuits [8], such as the hippocampus, anterior cingulate cortex, ventromedial prefrontal cortex, and amygdala. The hippocampus, a structure shown to contribute to PTSD etiology [9], [10], plays a role in the fear conditioning and extinction aspects of contextual memory [11], [12], as well as in stress regulation [13], [14]. Different meta-analyses revealed significantly smaller hippocampal volumes in PTSD patients than control subjects [15]–[17]. It has been suggested that the ventromedial prefrontal/anterior cingulate cortex, and the amygdala take part in a model of PTSD pathogenesis in which the cortical regions fail to inhibit a hyperactive amygdala [18], [19]. A meta-analysis of structural abnormalities in PTSD found significantly smaller amygdala volumes in adults with PTSD compared with both healthy and trauma-exposed controls [15]. Recent structural studies found that ventromedial prefrontal/anterior cingulate cortex are reduced in PTSD subjects [20]–[26].

Studies of the neurobiology of PTSD had focused on a narrow spectrum of trauma. Indeed, combat veterans and survivors of childhood physical and sexual abuse accounted for 85% of all subjects recruited to PTSD neuroimaging studies [27]. Thus far, no study has investigated the structural brain changes associated with PTSD elicited by urban violence. Urban violence is an important and pervasive cause of human suffering, especially in the large population centers of the developing world [28]. Violent events, such as armed robbery, are very frequent in certain cities, and these episodes increase the risk of developing PTSD [29]. In fact, an epidemiologic study has revealed that being threatened with a weapon accounted for 32.6% of the risk of PTSD development in women [2].

Assaultive trauma is characterized by forceful invasion of the peripersonal space, which is defined as a margin of safety around the body [30]–[32]. The invasion of this margin of safety is often experienced as a threat to an individual's psychological or biological integrity and may lead to intense discomfort and anxiety [30]–[33]. A number of functional studies in PTSD have shown altered activity in premotor cortical areas [34]–[37]; these areas are involved both in preparing to respond to a threatening situation and in peripersonal space representation [38]–[41].

A recent structural study in PTSD victims of child sexual/physical abuse, a traumatic event that involves forceful invasion of personal space, observed volume reduction in premotor cortical region [25]. Structural abnormalities in hippocampus, anterior cingulated/ventromedial-prefrontal cortex and amygdala, but not in premotor areas, were observed in studies assessing traumatic situations such as terrorism [42], disaster [20], [43], [44], war [21]–[23] and disease [45]. These traumatic situations can be very heterogeneous and do not necessarily involve an invasion of peripersonal space.

Recently, an experimental study provided evidence that life-threatening urban violence events are a major trigger for motor defensive reactions in humans [46]. Neurobiology studies have suggested that the premotor cortex incorporates both a representation of peripersonal space and defensive-like motor repertories [38]–[41]. Could PTSD resulting from forceful invasion of peripersonal space be associated with structural alteration in cortical premotor areas? In this study, we address this question by examining PTSD patients exposed to the trauma of urban violence. We expect to observe a volumetric reduction in the premotor cortex in these particular patients, in addition to the structural alterations in the hippocampus, anterior cingulate/ventromedial-prefrontal cortex and amygdala that are typically observed in PTSD patients.

## Materials and Methods

### Participants

In total, 32 patients with current PTSD were recruited from an outpatient university clinic that specialized in the posttraumatic stress assessment and treatment of urban violence victims. The diagnosis of PTSD was obtained using the Structured Clinical Interview for DSM-IV Axis I [47], which had previously been translated and adapted to Portuguese [48]. For the following reasons, 16 patients were excluded from the study: history of alcohol/substance dependence or abuse (n = 9); psychosis (n = 2); risk of suicide (n = 1); claustrophobia (n = 3); and bullet lodged in the head (n = 1).

We acquired structural magnetic resonance imaging scans of the remaining 16 patients. These patients were under pharmacological treatment with antidepressant drugs in adequate doses according to the recommended guidelines for PTSD [49] and presented major depression co-morbidity. The control group was selected from a list of approximately 300 employees of the Federal University of Rio de Janeiro. From this list, 21 victims of urban violence matched by age, education level and gender with the patients were selected for an interview. After the administration of the Structured Clinical Interview for DSM-IV Axis I, 4 participants were excluded because they presented a past history of PTSD (n = 2), obsessive compulsive disorder (n = 1), or depression (n = 1). The remaining 16 trauma-exposed participants were scanned as controls. They met criteria A1 and A2 (DSM-IV) and had no past or current history of mental disorder. The predominant traumatic event for both patients and controls was armed robbery. The characteristics of the sample are described in Table 1.

**Table 1:** Characteristics of the sample population.

Characteristics	PTSD		Controls	
	N	%	N	%
Gender				
Women	9	56.2	9	56.2
Men	7	43.8	7	43.8
Relationship status				
Single	1	6.3	2	12.4
Married/living with partner	12	75.0	13	81.2
Divorced/widower	3	10.8	1	6.3
Type of traumatic event				
Armed violence	13	81.2	12	75.0
Motor vehicle accidents	3	10.8	2	12.4
Assault without gun	-		1	6.3
Sexual abuse	-		1	6.3
	Mean	SD	Mean	SD
Age	43.3	5.78	44.9	6.60
Education level (years)	10.5	2.59	11.8	3.56
Time elapsed since trauma (in years)	3.0	4.8	11	9.8

### Ethics statement

This study was approved by the Ethics Review Board of the Institute of Psychiatry of the Federal University of Rio de Janeiro. Written informed consent was obtained from all of the participants after a detailed description of the study.

### Psychometric Assessment

All subjects included in our study were victims of urban violence. To characterize the contrast between the test group of patients with PTSD and the control group of trauma-exposed individuals without PTSD, an analysis of PTSD symptomatology, affective dispositions and resilience traits was conducted. PTSD symptom severity was assessed using the Post-Traumatic Stress Disorder Checklist - Civilian Version (PCL-C) [50], translated and adapted to Portuguese by [51]. The PCL is a standardized self-report rating scale for PTSD and is composed of 17 items that correspond to the key symptoms of PTSD. The participants indicate how much they have been bothered by a symptom over the past month using a 5-point scale that ranges from 1 (Not at All) to 5 (Extremely). Affect traits were assessed using the Positive and Negative Affect Schedule scale (trait version, PANAS-T) [52], which uses 10 positive and 10 negative adjectives describing mood. The participants rated each mood adjective on a scale from 1 (very slightly or not at all) to 5 (extremely). The Ego-Resilience scale (ER-89) [53] was used to assess how each individual subject manages the challenges and experiences of daily life. The scale has 14 items, each of which are rated on a scale from 1 (does not apply at all) to 4 (applies very strongly).

### MRI acquisition

All participants were scanned at the LABS-D'Or Network outpatient MRI unit using a 1.5-Tesla MR scanner (Philips Medical Systems, the Netherlands). High-resolution structural T1-weighted volumetric images were acquired with full head coverage. In total, 160 contiguous sagittal slices were acquired for each participant, using the following parameters: TR = 8.11 ms, TE = 3.7 ms, flip angle = 8°, field of view = 256 mm, slice thickness = 1.00 mm.

### Pre-processing of images

Voxel-based morphometry (VBM) was conducted using SPM5 statistical parametric mapping software (Wellcome Department, University College London; http://www.fil.ion.ucl.ac.uk/spm/) running in MATLAB 7 (Mathworks, Sherborn, MA). The images were segmented by gray matter (GM), white matter (WM) and cerebrospinal fluid (CSF). Next, we applied the DARTEL toolbox for normalization of the data. This methodological approach provided improved inter-subject alignment accuracy [54].

DARTEL warps images from individual subjects to the group template, and an initial group average is created as the beginning target with rigidly aligned images. Then, individual images are warped to match the average. After a few iterations of warping, a shaper average is created. This procedure was repeated six times. At each stage, the regularization is reduced; hence, the amount of warping increases at each iteration. Additionally, Jacobian-scaled (“modulated”) warped tissue class images were created to conserve the total volume within the voxels. Then, segmented, normalized and modulated GM images were smoothed with an 8-mm FWHM Gaussian kernel. The data were subsequently warped to MNI space.

### Statistical Analyses

We compared psychometric measures between PTSD and controls groups using Student's t-test for independent samples. Statistical comparisons of gray matter volume between PTSD and controls groups were performed using t-tests with statistical parametric mapping (SPM5). Total brain volume was treated as a confounding variable. The resulting set of voxel values for each contrast constituted a statistical parametric map of t-statistics SPM(t). The SPM(t) values were converted into z-scores SPM(z). Significance was set at a voxel level of p<0.05, which was FDR-corrected for multiple comparisons.

Additionally, as the hippocampus and amygdala have been the focus of many studies in PTSD, we performed a region of interest (ROI) analysis restricted to these structures. These ROIs were defined using the Wake Forest Pickatlas, version 1.04 [55], and the analysis was performed using the Marsbar toolbox (http://marsbar.sourceforge.net/) for SPM5.

## Results

The PTSD group presented significantly greater symptom severity scores than the control group (p<0.0001). In addition, psychometric analysis indicated that the PTSD group had lower positive affect scores (p<0.0001) and higher negative affect scores (p<0.0001) than the control group. Moreover, the PTSD subjects had lower ego-resilience scores than the control subjects (p<0.0001).

**Figure 1:**
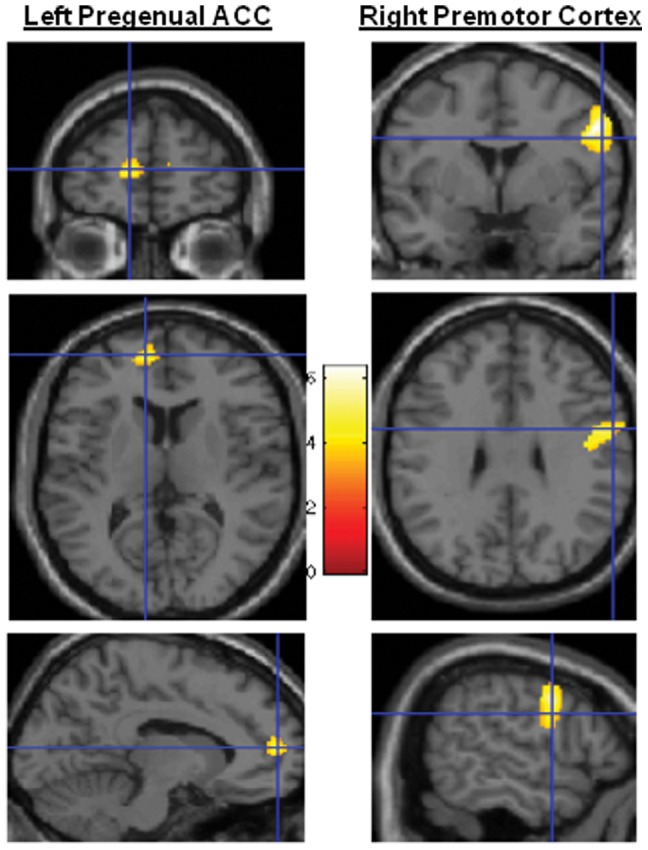
Statistical parametric mapping (SPM) showing clusters in the left pregenual anterior cingulate cortex and the right ventral premotor cortex. There are significant differences in gray matter volume between the PTSD and control groups.

VBM analysis revealed decreased gray matter volume in the premotor cortex of the PTSD group compared to the traumatized control group, p<0.05 corrected. We also observed a gray matter volume reduction in the anterior cingulate cortex in PTSD subjects, p<0.05 corrected (Table 2 and Figure 1). Neither the hippocampus nor the amygdala differed in volume between the PTSD and control groups.

**Table 2:** Results of t-tests comparing PTSD and control groups.

Brain region	Laterality	Coordinates			Cluster size	Z-score
		X	Y	Z		
Ventral premotor cortex*	R	53	−1	35	975	5.01
Pregenual anterior cingulate cortex*	L	−12	54	10	242	4.14
Superior parietal sulcus†	R	31	−76	47	83	4.16
Superior temporal sulcus†	R	53	−25	−3	52	3.37

Stereotactic coordinates are quoted within MNI space.

* p<0.05 corrected,

† For a more lenient statistical significance threshold of p<0.001 (uncorrected).

We estimated the cluster at the anterior cingulate cortex to be centered at the pregenual BA 32, according to the subdivision of the cingulate described by Vogt [56]; and the cluster at the premotor cortex to be centered at ventral BA 6, according to the subdivision of motor areas described by Rizzolati et al [40].

ROI analysis showed that the hippocampal and amygdala volumes of traumatized victims with PTSD did not differ from controls.

## Discussion

The goal of this study was to investigate gray matter volume alteration in PTSD victims of urban violence, a traumatic event characterized by forceful invasion of peripersonal space. We found reduced gray matter volume in the ventral premotor cortex in PTSD patients compared to traumatized controls. We also found a reduction in the anterior cingulate cortex.

The present results suggest that structural alteration in the ventral premotor cortex may contribute to PTSD pathophysiology in victims of interpersonal violence. Graziano and Cooke [39] showed that the premotor cortex is involved in protective mechanisms that are essential in extreme, life-threatening situations [39]. These authors suggested that this region is involved in the representation of the peripersonal space as a margin of safety around the body, as well as in the selection and coordination of defensive behavior [39]. Studies of social interactions have indicated that individuals instinctively dynamically regulate the interpersonal distance between themselves and others to avoid discomfort [57] and that the invasion of peripersonal space can lead to anxiety on the part of the victim [33].

Premotor cortex structural abnormalities observed in PTSD patients may be associated with inappropriate construction of a margin of safety around the body, as well as inefficient selection and coordination of defensive responses. Restoring the margin of safety around the body in the aftermath of violence may be an essential aspect of the recovery from these traumatic events. The integrity of the premotor cortex is likely a critical factor for these restorative mechanisms.

Structural abnormalities in premotor cortex observed in PTSD patients may be associated to inappropriate construction of a margin of safety around the body as well as inefficient selection and coordination of defensive responses. Restoring the margin of safety around the body in the aftermath of violence may be an essential part of the recovering processes from these traumatic events. The integrity of the premotor cortex is likely important for these restorative mechanisms.

We observed significantly gray matter volume reduction in the anterior cingulate cortex in PTSD patients compared to traumatized controls. Previous VBM studies also observed volumetric reductions in this region in PTSD patients [20]–[25].

A review of functional imaging studies in PTSD detected less activation in this region as well as more activation in amygdala in response to emotional stimuli in patients compared to participants without PTSD [58]. These functional results are in accordance with a current model for PTSD; this model proposes that the anterior cingulate is hyporesponsive and that the amygdala is hyperresponsive in PTSD patients [58].

The anterior cingulate cortex is functionally and anatomically complex and heterogeneous [56], [59]. Vogt [56], based on cytoarchitectonic, connectivity and neurotransmitter receptor criteria, proposed a subdivision of the anterior cingulate cortex into subgenual and pregenual regions. The subgenual region is heavily connected to the amygdala in both humans and non-human primates [60], [61]. This region is also critical for the retention of fear extinction in healthy individuals [62] and is related to fear extinction deficits in PTSD patients [63]. The pregenual subdivision is less well connected to the amygdala [60], [61], suggesting that it plays a smaller role in fear circuit modulation. In the present study, we observed volume reduction in the pregenual subdivision of the anterior cingulate; one interpretation for the abnormalities observed in the pregenual subdivision of the anterior cingulate cortex is the following line of reasoning. Functional studies in healthy subjects revealed increased activity in the pregenual anterior cingulate cortex when experiencing feelings of happiness that are induced by words [64], films [65], recalled experiences [66]–[68], music [69] or faces [70]–[72]. A recent study using a sensitive meta-analytic method that analyzed a substantially large number of neuroimaging studies also found that the pregenual anterior cingulate cortex was consistently associated with happiness [73]. It seems reasonable to hypothesize that reduced gray matter volumes in the pregenual anterior cingulate cortex of PTSD patients could be associated with a reduced ability to experience pleasurable emotions. Indeed, impairment in the processing of pleasant cues in PTSD has been reported [74]. In the present study, psychometric assessments revealed that compared with trauma-exposed controls, PTSD patients scored significantly lower on both positive affect and ego-resilience scales, which measure two important components of the concept of happiness [75].

A meta-analysis of structural brain abnormalities in PTSD found significantly smaller hippocampal volumes in PTSD compared to controls with and without trauma exposure [15]. Here, we did not observe hippocampal volume reduction in PTSD patients compared to trauma-exposed controls, both in the whole brain and in the region-of-interest analyses. More studies are necessary to clarify whether the absence of hippocampus reduction in the present study is due to distinctiveness of urban violence trauma or to other characteristics of the sample and/or methodology.

Amygdala volume did not differ between PTSD patients and trauma-exposed controls. A meta-analysis of structural brain abnormalities observed significantly smaller left amygdala volumes in adults with PTSD compared with both healthy and trauma-exposed controls [15]. A subsequent meta-analysis on amygdala volume in adult PTSD patients showed no significant differences between PTSD and controls [76]. The authors of the more recent of these two meta-analyses [76] attributed this discrepancy to the inclusion of pediatric data in the initial meta-analysis [15].

As far as we know, this study is the first to find gray matter volume reduction in the ventral premotor cortex in PTSD patients. This finding may be explained by the type of traumatic event. Here, more than 80% of PTSD patients reported armed robbery as the index trauma. In this type of trauma, the victims were confronted with a threat represented by a weapon pointed at them, and their peripersonal space was violated by the robber. Other VBM studies assessed traumatic situations such as terrorism terrorism [42], disaster [20], [43], [44], war [21]–[23] and disease [45]. These traumatic situations, as well as those that assessed populations that experience mixed trauma, can be very heterogeneous and do not essentially involve an invasion of peripersonal space. Interestingly, a VBM study that evaluated victims of physical/sexual abuse, which also involves forceful invasion of the personal space, observed abnormalities in the supplementary motor area, a region adjacent to the ventral premotor cortex [25]. More studies evaluating traumatic events that violate peripersonal space are necessary to investigate motor area involvement in PTSD pathology.

### Limitations

Our study has a number of limitations. First, the sample size is relatively small. Second, the study lacks a second control group of healthy subjects that were not exposed to trauma. Third, this study is essentially cross-sectional and does not allow for causal conclusions to be derived regarding the structural alterations observed herein, which could have resulted from either PTSD or a pre-trauma vulnerability factor. Longitudinal studies will be essential for addressing this question.
